# Combined Surgical and Restorative Procedures to Treat Maxillary Canine with Gingival Recession and Cervical Wear

**DOI:** 10.1155/2022/2670994

**Published:** 2022-06-08

**Authors:** Dler Ali Khursheed, Faraedon Mostafa Zardawi

**Affiliations:** Department of Periodontics, University of Sulaimani, Sulaymaniyah, Kurdistan Region, Iraq

## Abstract

**Introduction:**

Gingival recession (GR) with cervical tooth wear is a major concern for patients on the prominent maxillary canines, from both esthetic and dentine hypersensitivity points of view. Hypersensitivity could be treated nonsurgically; however, esthetics remain the major patients' concern that mostly requires surgical intervention for covering the denuded root surface. Several surgical procedures are applied successfully for covering single and multiple gingival recession; however, these procedures are sensitive procedures and not always predictable. Semilunar coronally repositioned flap (SCRF) is a very simple procedure that found to be very predictable for covering a single recession in presence of sufficient keratinized gingiva apical to the recession. The procedure provides better clinical outcome by involving less adjacent papillary tissue that maintains greater blood supply and achieves maximum flap stability with mattress sutures with minimal postoperative complications. Therefore, this case report is aimed at explaining the simplicity of this surgical procedure in the presence of wide keratinized tissue around the recession and starting the restorative procedure after a sufficient soft tissue healing period. *Case Presentation*. 42-year-old systemically healthy female patient referred with a single wide gingival type 1 (RT1) and cervical wear around tooth #43. A semilunar coronally repositioned flap was released and advanced coronally to cover the denuded root totally; then, the flap stabilized by three mattress sutures, and complete root coverage was achieved. Six months later, the cervical lesion was restored with composite filling material. Sixteen-month and 32-month postoperative follow-up revealed full coverage of the denuded root surface with firm stable gingiva; later, the gingiva in the area looked stable and healthy.

**Conclusion:**

Using SCRF in treating RT1 recession in the presence of wide keratinized gingiva is very promising surgical intervention for receded root coverage that requires less technical skill.

## 1. Background

Gingival recession is defined as the shift of the free gingival margin apical to the cementoenamel junction [1]. Localized attachment loss with GR is very common on the facial surfaces of teeth in patients with high standards of oral hygiene care [2]. Treatment approaches for cervical tooth wear with GR can be nonsurgical, surgical, or combined surgical-restorative [3]. Gingival recession is treated and based on esthetic and functional consideration either nonsurgically by using topical desensitizing agents to reduce dentine hypersensitivity, or in cases of esthetic demands, gingival recession is managed by advanced coronal or lateral pedicle flaps combined with free connective tissue graft. In the current case, the recession was treated by advanced partial flap with semilunar incision with slight variation in clinical outcome [4, 5].

Probiotics were used to prevent cervical tooth decay and further recession due to gingivitis or periodontitis, which are considered as the primary cause of gingival recession [5].

The major goal of periodontal plastic surgery is to achieve complete root coverage with an esthetic outcome, rendering a thicker gingival biotype to maintain long-term stability [6, 7]. Subepithelial connective tissue graft (SCTG) is regarded as the most predictable root coverage procedure in single and multiple GRs [8]. However, SCRF may have advantages over SCTG in terms of simplicity and avoidance of two surgical procedures and tissue morbidity. Moreover, its predictability and esthetic results are considered promising. The following benefits are considered for SCRE, such as lack of tension on the flap or changes in the vestibular depth; further, the papillae mesial and distal to the tooth being treated remain intact from an esthetic point of view provides greater blood supply to the flap [9]. This case report attempts to show the potential of SCRF in respect to esthetic outcome, healing time, and patient comfort during the healing period.

## 2. Clinical Presentation

**Figure 1:**
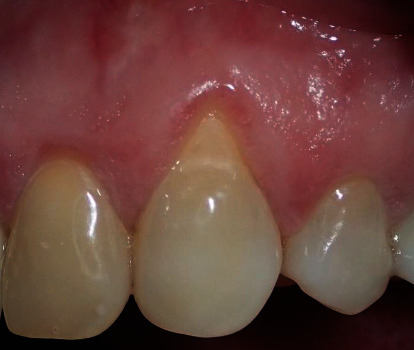
Tooth #43 class I Miller recession with root exposure and abrasion.

**Figure 2:**
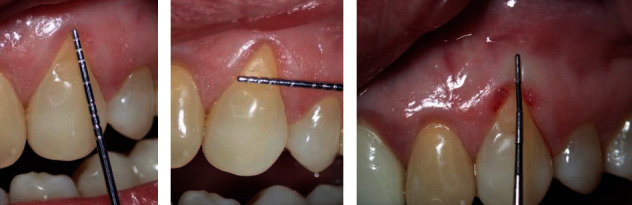
(a, b) Vertical and horizontal dimensions of the recession using Michigan probe. (c) Measuring the amount of attached keratinized gingiva around the recession by using Marquis periodontal probe.

A 42-year-old nonsmoker female patient attended the dental clinic complaining of tooth sensitivity and elongation of tooth number #43 (Figure 1). RT1 of 4 mm with cervical wear was detected on clinical examination of # (Figures 2(a) and 2(b)). Probing depth at the mesial, midlabial, and distal thirds facially measured 1 mm, respectively. A wide band of keratinized tissue was existed adjacent to the recession, and about 3 mm of attached keratinized gingiva was found apical to the recession (Figure 2(c)). Periodontally and systemically, the patient was fit for surgery.

Before performing the surgical procedures, the patient was provided with an information sheet including the benefits, risks, alternative treatments, and consequences of nontreatment which were discussed in detail with the patient and a signed consent was achieved from the patient. Further, the study was registered at the Scientific Committee of College of Dentistry (N 24 at 3^rd^ Sept. 2019), and ethical approval was obtained from the ethical committee of University of Sulaimani.

The patient then signed a consent form.

## 3. Case Management

The patient was instructed to rinse with 0.2% chlorhexidine gluconate mouthwash before the operation. Local anesthesia was given to the area, and the semilunar flap was designed based on the amount of keratinized gingiva at either sides and apical to the recession.

**Figure 3:**
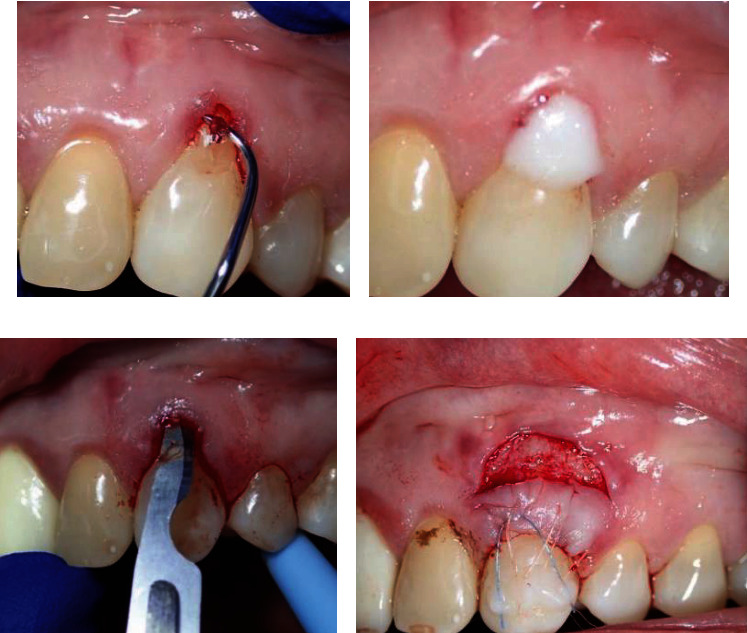
(a) Root planing using sharp Gracey curette. (b) Root detoxification using EDTA gel for 2 minutes. (c) Using 15C blade to make partial thickness flap. (d) The coronally repositioned semilunar flap was secured by three horizontal mattress sutures and fixed with flowable composite to the crown of the tooth.

The exposed root was properly instrumented with a Gracey 3/4 curette and frequently irrigated with normal saline (Figure 3(a)). The root was then conditioned with EDTA gel for 2 minutes, followed by profound irrigation (Figure 3(b)). Using a 15C blade, sulcular incision of about 3 mm was performed laterally and apically to raise a partial thickness flap. The apical margin of the partial thickness flap was excised to make a semicircular flap (Figure 3(c)). The adjacent interdental gingival papillae were maintained with lesser undermining to provide the flap with maximum blood supply. The flap was further advanced coronally and fixed with 3 superficial horizontal mattress sutures, and the wound was covered with periodontal dressing to provide further support and protection from the external environment. The sutures were held by flowable composite on dry, etched, and bonded enamel (Figure 3(d)). Antibiotics and analgesics were not prescribed. She was also instructed to not brush the surgical area for at least two months.

## 4. Clinical Outcomes

**Figure 4:**
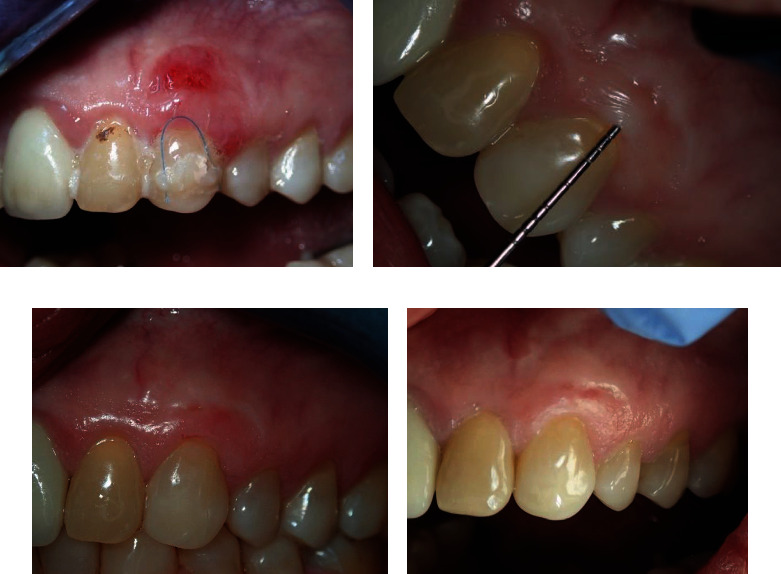
(a) Ten days after removing the periodontal dressing and sutures. (b) Two months later, the gingiva was very stable and showed resistance to the periodontal probe. (c) At six months, the gingiva remained very stable with small amount of scar tissue on the donor area and was filled with composite filling. (d) 32 months postoperatively, the gingiva looked very stable and healthy.

After 10 days, the periodontal dress and sutures were removed, and the recession was fully covered (Figure 4(a)). No surgical recession was observed after 10-day, 20-day, one-month, and two-month follow-ups (Figure 4(b)). The donor area was red and covered with granulation tissue after 10 days and more scar-like at 20 days. The tissue was firmly attached to the root surface with 1 mm sulcular depth at two-month and 6-month follow-up time (Figure 4(c)). The complete tissue remodeling was seen, and the cervical wear was restored with adhesive filling material. Sixteen months later, the gingiva and the restoration looked very stable (Figure 4(d)).

## 5. Discussion

Semilunar advanced coronal flap is a simple and predictable procedure for treatment of RT1 when a wide band of keratinized gingival is present apical to a recession [9]. The major differences between SCTG and SCRF are that the SCTG blood supply comes from the inner surface of stretched alveolar mucosal tissue and underlined periosteum, while the SCRF is a bilateral pedicle graft. It has profound collateral blood supply from interdental gingival tissues because the interdental papillae remain intact [10]. Additionally, a larger area of lateral papillary connective tissue remains undissected with SCRF.

Further, the procedure is simple and requires less time to be performed with less postoperative swelling and bleeding compared to other root coverage procedures.

EDTA was applied to render a clean root surgical interface free of any bacterial products and residual smear layer to enhance interaction between root surface collagen fibrils and connective tissue cells [11, 12], and therefore, higher penetration resistance was shown to probing the recession site after two months, which could be attributed to proper integration of connective tissue with root dentine. At this stage, the cervical lesion was restored to achieve an integrated soft and hard tissue between the restored cervical lesion and color and position and consistency of the gingiva at the area of the recession 6 months postoperatively.

The procedure first published by Tarnow in Journal of Clinical Periodontology 1986. It was found to be a successful method for covering a single recession without suturing the flap, when sufficient zone of keratinized gingiva is present apical to the recession [10].

There is no fundamental modification or novelty performed in this study; however, the procedure was simplified by a small partial thickness flap without lateral extension with semilunar incision at the apical area, and the keratinized tissue was retracted and fixed coronally with composite by three superficial horizontal mattress sutures (Figures 3(a)–3(d)).

Keratinized gingiva was retracted coronally to cover the recession and remain stable, immobilized during the healing period. The procedure is now minimally invasive with less postoperative patient complaints and better clinical outcome for a single root coverage surgical procedure with similar color and texture of gingival tissue specially the area is an esthetic zone such as maxillary canine.

After complete heeling, the survival tooth wear was treated by composite restoration to avoid further tooth wear of cervical dentine and future recession at the area. After thirty-two months postoperatively, still full coverage of the denuded root is achieved and maintained (Figure 2(d)).

## Data Availability

All data are available on demand that are already included in the manuscript.

## Additional Points

*Key Finding*. Semilunar coronally positioned flap is an appropriate choice to treat gingival recession in the presence of wide keratinized gingiva. *Why is this case new information?* Semilunar flap is aneffective and simple procedure choice to restore the defect in the presence of thick gingival biotype around the recession. Suturing techniques provide maximum stability to the graft. *What are the keys to successful management of these cases?* Maintaining maximum blood supply to semilunar flap from collateral blood supply by involving less of the adjacent interdental tissues. Presence of thick and wide gingiva apically and interdentally. *What are the primary limitations to success in these cases?* Amount of keratinized tissue around the recession and presence of receded adjacent interdental papillae. Apical and lateral extensions of the recession. Healing may show scare tissue at the site of the incision.
